# Peritoneal Tuberculosis and the Associated Diagnostic Challenges in Gynecology: A Case Report

**DOI:** 10.7759/cureus.77250

**Published:** 2025-01-10

**Authors:** Patrícia Nazaré, Francisco Vale, Leonardo Carneiro, Ana Tomé, Inês Gomes

**Affiliations:** 1 Gynecology and Obstetrics, Hospital Garcia de Orta, Almada, PRT; 2 Infectious Diseases, Hospital Garcia de Orta, Almada, PRT; 3 Pathology and Laboratory Medicine, Hospital Garcia de Orta, Almada, PRT

**Keywords:** ascites, laparoscopy, peritonitis, tuberculosis, tuberculous peritonitis

## Abstract

Peritoneal tuberculosis should be considered in young patients presenting with non-specific abdominal pain, constitutional symptoms and ascites. Here, we describe a case of a 29-year-old woman with pelvic pain, abdominal distension and weight loss. She presented with a distended, painful abdomen, and painful mobilization of the uterus and adnexa. Imaging revealed multiloculated adnexal formations, ascites and peritoneal thickening. A diagnostic laparoscopy was performed, with findings suggestive of peritoneal carcinomatosis, but the biopsies were compatible with granulomatous peritonitis. The definitive diagnosis was confirmed by cultural growth of *Mycobacterium tuberculosis*, so the patient started antituberculosis treatment. This case illustrates the difficulty in establishing the differential diagnosis between peritoneal carcinomatosis and granulomatous peritonitis.

## Introduction

Globally, a total of 8.2 million people were reported as newly diagnosed tuberculosis (TB) cases in 2023, up from 7.5 million in 2022 and 7.1 million in 2019 and far above the levels of 5.8 million in 2020 and 6.4 million in 2021 [1]. Peritoneal TB is a chronic infection caused by agents of the *Mycobacterium tuberculosis* complex (MTBC) and accounts for 6.1% of all extrapulmonary TB cases and 1%-2% of all tuberculosis cases [2-5]. Due to its rarity, epidemiological data are scarce.

Peritoneal TB (or tuberculous peritonitis) is the sixth most common form of extrapulmonary tuberculosis [2]. Peritoneal TB usually occurs due to hematogenous spread of bacilli from a primary pulmonary site to the peritoneum, but may also result from lesions in the adjacent organs such as the fallopian tubes, adnexa, or psoas abscess [6,7]. It can also occur through the ingestion of bacilli present in sputum, from a pulmonary focus, or consumption of contaminated dairy products [8]. Moreover, infected nodes can spread bacilli through the lymphatic system [7].

The disease is predominantly seen in young patients, with reported peak ages between 20 and 40 years. Although extrapulmonary tuberculosis is overall more prevalent among women than men (especially in endemic countries), peritoneal tuberculosis affects men and women equally, in developed countries [4,5].

The predominant clinical features of peritoneal TB are ascites (73%), abdominal pain (65%), body weight loss (61%), fever (59%) and abdominal tenderness (48%). Abdominal pain is usually non-localized and vague [8]. Other less frequent clinical signs/symptoms include constipation, diarrhoea, hepatomegaly and splenomegaly [9]. The clinical features of peritoneal TB evolve over a period of several weeks to months and may resemble chronic pelvic inflammatory disease or any other pelvic disorders including ovarian carcinoma [4,9]. Due to its variable and non-specific clinical presentation, it requires a wide range of differential diagnoses, including infectious, neoplastic, autoimmune conditions, other granulomatous diseases and other causes of ascites. Unfortunately, the diagnosis of peritoneal tuberculosis is difficult due to non-specific laboratory findings, so a combination of different diagnostic tests is used in order to reach a diagnosis. Paracentesis with fluid analysis and imaging may be helpful, but the microbiological analysis (and eventually molecular biological tests) of the biopsy tissue would be required for definitive disease diagnosis in most cases [6].

If this diagnosis is suspected, empirical medical treatment should be initiated, as delay in treatment may lead to worse outcomes. The first-line regimen consists of isoniazid, rifampicin, pyrazinamide and ethambutol, administered daily for two months, followed by four months of isoniazid and rifampicin [9]. There is no evidence to routinely extend it beyond six months [7,9]. The aim of this case report is to illustrate and highlight the challenges and particularities in the diagnosis of peritoneal tuberculosis.

## Case presentation

A previously healthy, nulliparous 29-year-old Black woman (born in Almada, Portugal) presented to the Emergency department complaining about persistent pelvic pain/discomfort, abdominal distension, dysuria and weight loss (4 kg) during the last eight months. She denied having fever, dysmenorrhea or abnormal vaginal discharge, but mentioned sporadic dyspareunia. Empirical antimicrobial therapy targeting pelvic inflammatory disease and urinary tract infection had already been prescribed several times (namely, ceftriaxone, doxycycline, metronidazole, cefuroxime), without clinical improvement. She mentioned to have regular menstrual cycles, without contraception, and a negative cervical cancer screening test (from 2022).

On physical examination, the patient was apyretic and normotensive, with low body weight (body mass index of 16 kg/m^2^) and revealed a poor clinical condition. The abdomen was diffusely distended and not fully depressible, slightly painful during palpation, but without palpable organomegaly. Speculum evaluation revealed a non-specific vaginal discharge and no cervical lesions; during bimanual palpation, she reported pain during cervical and adnexal mobilization, and palpable pelvic lesions were not detected.

**Figure 1:**
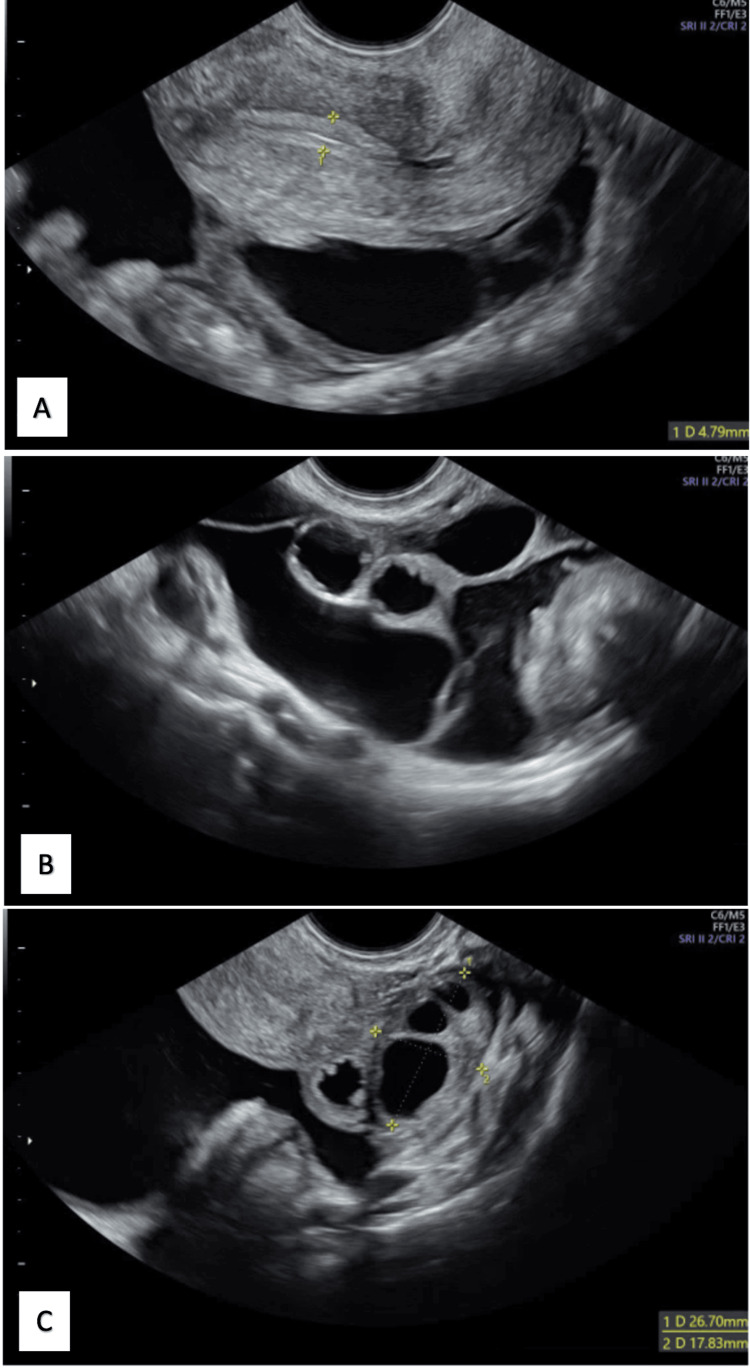
Transvaginal ultrasound evaluation before treatment (A) Uterus with a thin and regular endometrium, with apparently loculated anechogenic fluid, with thickened walls, in the pouch of Douglas. (B) Left adnexal area, with anechoic multiloculated cystic lesions, with hydrosalpinx, ascites and thickening of the peritoneum. (C) Right adnexal area, with an apparently normal ovary, is visualized.

Transvaginal ultrasound (US) was performed revealing a normal-sized uterus and bilateral anechoic, multiloculated cystic lesions, without solid component, measuring 81 x 73 mm in the left adnexal area, and 49 x 20 mm in the right adnexal area, with ascites and thickening of the peritoneum (Figure 1).

A laboratory evaluation revealed normal leukocytes (5100/μL) without neutrophilia (57.2%) and a slight elevation in the C-reactive protein level (2.23 mg/L). The levels of liver transaminases, urea, creatinine, and lactate dehydrogenase were normal. Human chorionic gonadotrophin was negative.

A pelvic computed tomography (CT) scan confirmed the previous imaging findings, highlighting that these aspects could be related to a pelvic inflammatory disease complicated by peritonitis, but not allowing the exclusion of carcinomatous ascites in the context of malignant adnexal pathology.

The patient was admitted in the Gynecology department for etiologic investigation, and empiric antimicrobial therapy was initiated with ceftriaxone, doxycycline and metronidazole. Although she denied having fever, it was observed during hospitalization that she had a tympanic temperature of 37.5-37.7ºC between 6 PM and midnight. Urine culture and sexually transmitted disease screening were negative. A slight increase in CA125 (42.7 U/mL, reference value <35 U/mL) and CA15-3 (60.4 U/mL, reference value <36.4 U/mL) was detected, with negative CEA and CA19-9.

Whilst in hospital, the patient remained stable, but with persistent pelvic pain. Hence, a diagnostic laparoscopy was performed that revealed lesions that were suggestive of disseminated peritoneal carcinomatosis, with small yellowish-white infracentimetic lesions, covering the entire peritoneal cavity, of variable sizes and regular contour, with adhesions that did not allow the individualization of the uterus and adnexa. Ascitic fluid and peritoneal biopsies were collected for cytological, histological, and microbiological studies. A total of 800 mL of ascitic fluid was collected. Adenosine deaminase (ADA) measured in ascitic fluid was 45.5 U/L (reference value <40 U/L) and total protein value was 6.6 g/dL.

Given the suspicion of peritoneal carcinomatosis, a chest CT scan, pelvic magnetic resonance imaging (MRI) and mammography were requested after surgery. The chest CT scan and mammography were normal. Pelvic MRI revealed an elongated hydric formation in the left adnexal region, measuring 8 cm, with a similar image in the right adnexal region measuring 4 cm, with no evident solid component, without suspicious characteristics, which could be related to bilateral hydrosalpinx (Ovarian-Adnexal Reporting and Data System, or O-RADS, MRI 2), without lymphadenopathies.

Ten days after surgery, the patient presented with the same complaints, a distended abdomen with ascites, maintaining the previous imaging and analytical findings (CA125, 36.6 U/mL; CA15-3, 75.9 U/mL).

**Figure 2:**
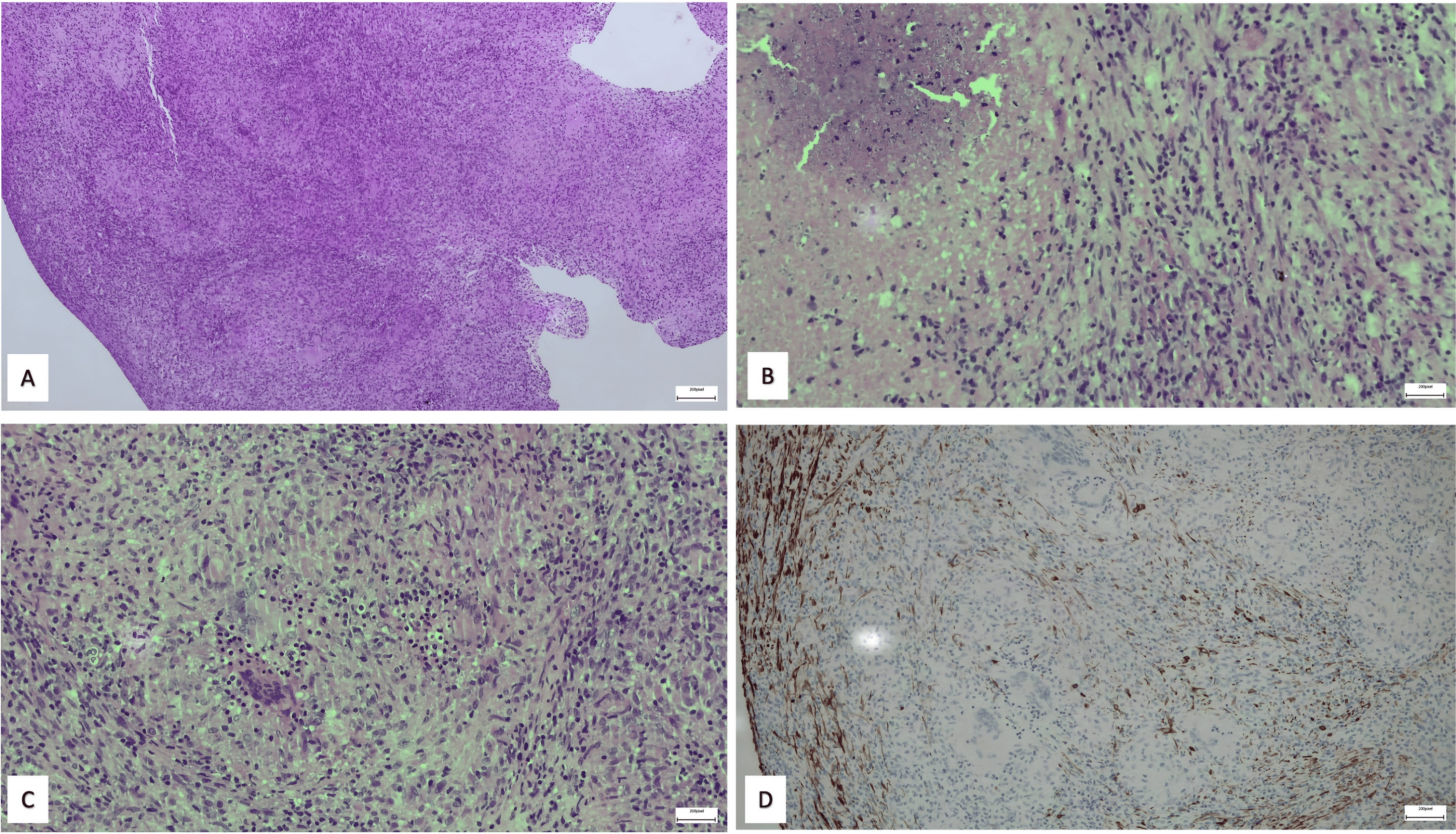
Histological examination indicating granulomatous peritonitis (A) The histological section is occupied by chronic histiocytic inflammatory tissue with granulomatous architecture. (B) Granulomatous inflammation with necrosis. (C) Multiple granulomas. (D) The tissue tested did not express cytokeratins detectable by the AE1/AE3 antibody cocktail.

Finally, peritoneal biopsy was negative for malignancy, as the cytology of the ascitic fluid was, but it revealed granulomatous peritonitis (Figure 2) with a positive culture (phenotypic test) for MTBC. These findings were diagnostic for peritoneal tuberculosis.

The patient was referred to the Infectious Diseases department, where she began treatment with first-line antituberculosis medications, including rifampicin, isoniazid, ethambutol, and pyrazinamide. The patient was tested for resistance to antituberculosis drugs, with sensitivity to first-line drugs (isoniazid, rifampicin, pyrazinamide, streptomycin and ethambutol).

When asked about epidemiological risk factors, she denied any prior respiratory symptoms or contact with individuals known or suspected to have tuberculosis. Her travel background was limited to a single trip to London and she always lived in Portugal. She lived with her brother and two nephews. The examination of the vaccination record revealed that Bacillus Calmette-Guérin vaccine had been administered during her childhood.

**Figure 3:**
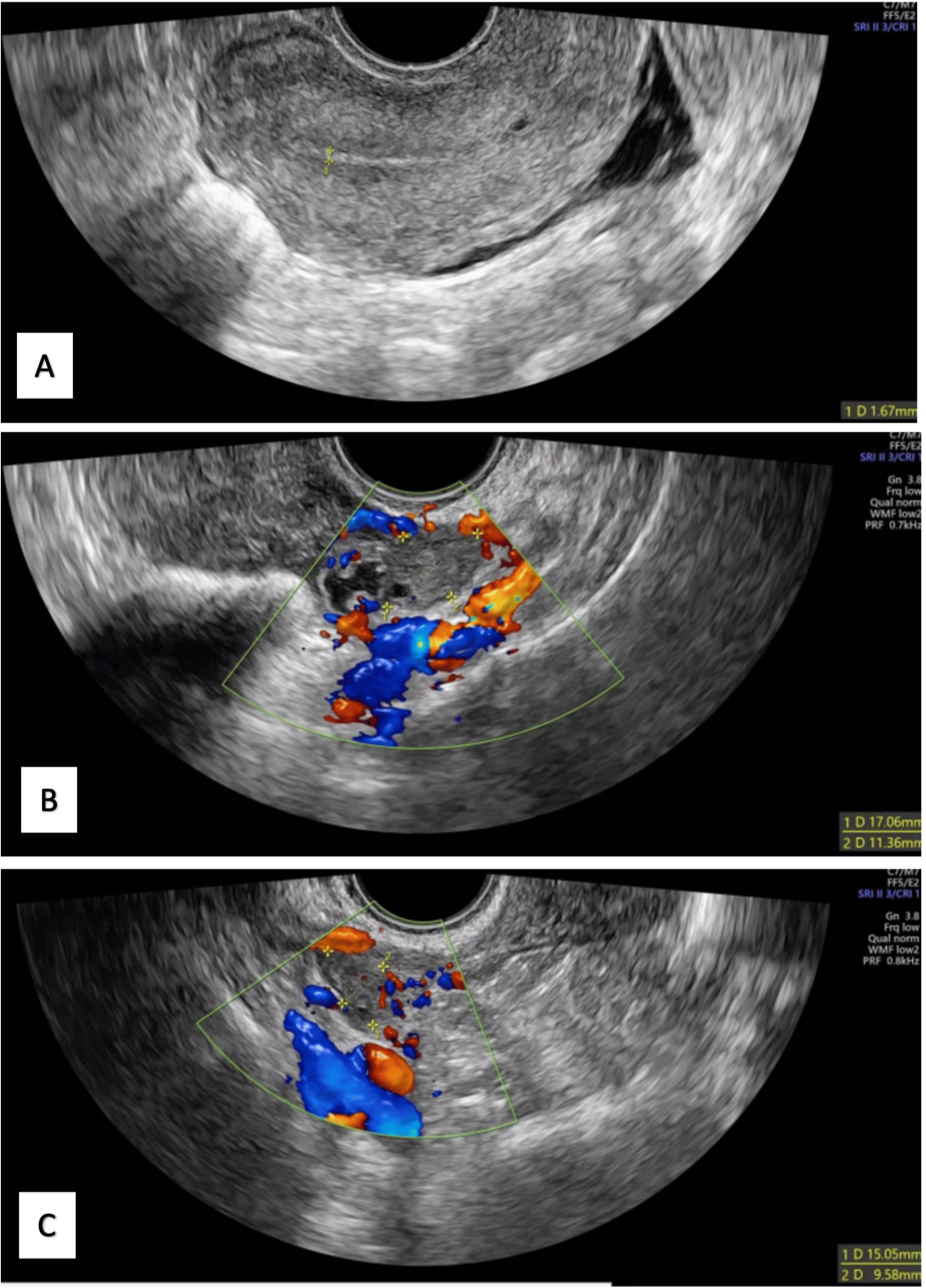
Transvaginal ultrasound revaluation one year after treatment with antituberculous drugs (A) Uterus with a thin and regular endometrium, and a thin layer of free fluid in the pouch of Douglas. (B) Right adnexal area with a normal ovary and mild hydrosalpinx. (C) Left adnexal area, with the left ovary and without the anechoic multiloculated cystic lesions previously described.

In the early stages of treatment, several evacuation paracentesis procedures were performed for symptomatic relief. After two months of quadruple therapy and showing favourable clinical and imaging improvements, along with confirmation of drug susceptibility, the regimen was adjusted to rifampicin and isoniazid. When six months of treatment had been completed, an abdominopelvic CT scan was again requested, showing a tubular image in the pelvic cavity, involving the uterus with an extension of approximately 10 cm and a thickness of 2 mm, compatible with loculated ascites or hydro/pyosalpinx. Given these imagological findings, it was decided to prolong antituberculosis therapy until completing nine months of treatment. By the end of the treatment, the patient had no symptoms and a near-complete regression of imaging abnormalities was observed (Figure 3), with a normalized CA125 level of 6.3 U/mL.

## Discussion

Tuberculosis remains a public health problem worldwide. In Portugal, according to the latest tuberculosis surveillance and monitoring report, in 2022, 1518 cases of TB were detected, with a notification rate of 14.5 per 100,000 inhabitants [10].

The diagnosis of extrapulmonary TB can be challenging, requiring a high index of suspicion. It presents with non-specific clinical and radiological features. In many cases, no clear epidemiological risk factors can be identified and only 15%-25% of patients with abdominal TB have concomitant active pulmonary disease [2,4,7,8]. Qian et al. found that patients aged ≥45 years, female patients, those infected with human immunodeficiency virus, and suffering from end-stage renal disease were at a significantly elevated risk of extrapulmonary TB [11]. In this case, being female was the only risk factor present. Additionally, there was no history of tuberculosis or any known exposure to suspected cases. Later, it was also confirmed that there was no sign of actual or previous pulmonary disease. All these factors made the diagnosis even more challenging.

In this case, ascites, abdominal pain, and weight loss were substantial, which corresponds to the most common clinical manifestations reported in the literature. Considering that a tympanic temperature above 37.7ºC is considered a fever, our patient was in a subfebrile state, which is consistent with the presence of an inflammatory or infectious process.

Laboratory evaluations reveal non-specific findings, most often mild normocytic normochromic anaemia, elevated erythrocyte sedimentation rate (usually not exceeding 60 mm/hour), thrombocytosis and hypoalbuminemia. The WBC count is usually normal, but lymphocytosis is common [2,6]. This patient had a nearly normal initial laboratory evaluation, with only a slight elevation in the C-reactive protein level, which is not very common in usual acute pelvic inflammatory disease. This is yet another point in favour of the diagnosis of peritoneal tuberculosis, a chronic inflammatory process.

CA125 levels are elevated in almost all patients with ascites, including those having peritoneal tuberculosis, pelvic inflammatory disease, endometriosis, fibroids or hepatitis [4,6]. However, any woman who presents with a pelvic mass, ascites and increased serum CA125 levels must be considered to have ovarian cancer until proven otherwise [4]. The patient presented with these characteristics (despite the very slight elevation in CA125), so it was essential to rule out the possibility of ovarian carcinoma, which is why a diagnostic laparoscopy with biopsies was performed.

Tuberculin skin testing and interferon-gamma release assays may be supportive, but cannot differentiate between latent and active TB [2].

The ascitic fluid, which can be collected by paracentesis or laparoscopy, should be analysed in terms of macroscopic aspect, cell count, cytological study, Gram staining and Ziehl-Neelsen stain, microbiological cultures, ADA level and, if available, nucleic acid amplification test (NAAT) for mycobacteria [9].

Frequently, the colour of ascitic fluid is light yellow (cloudy, chylous or hematic appearances are less frequent) and the cell count usually contains 500-1500 WBC/mm^3^, predominantly lymphocytes, but other patterns, including neutrophilic predominance, can be seen [2]. The cytological study aims to identify neoplastic cells since carcinomatous ascites are one of the differential diagnoses [9]. The sensitivity and specificity of ADA levels in ascitic fluid detecting peritoneal TB can be as high as 100% and 97%, respectively, when cut-off values of 36 to 40 IU/L are used [12]. Other measurements, such as ascitic fluid lactic dehydrogenase, serum ascites albumin gradient or glucose, are not recommended for the diagnosis [6,9].

Up to 25%-45% of the patients with peritoneal TB present with abnormalities on the chest radiograph [8]. The majority of patients do not have radiological signs, as seen in our patient.

Sonographic findings that are suggestive of peritoneal TB include localized ascites, septations, fine strands and lymphadenopathy with hypoechogenic centres indicating caseous necrosis. Although neither necrosis nor calcification of lymph nodes is specific of TB, they are highly suggestive of this disease [8,13]. A regular and uniform thickening of the peritoneum with pronounced enhancement on a CT scan suggests peritoneal TB, whereas nodular implants and irregular peritoneal thickening suggest carcinomatosis. US and abdominal CT scan provide different information, so they may be complementary to each other: US is superior to a CT scan in revealing the multiple, fine, and mobile septations, while a CT scan can highlight the peritoneal, mesenteric or omental involvement [6].

MRI is a more time-consuming and expensive examination that may be used to avoid the radiation exposure in young patients or when intravenous contrast is not available or is contraindicated. Nevertheless, it is less sensitive than a CT scan in detecting lymph node calcifications [8]. While MRI is the gold standard test when pelvic malignancy is suspected, a CT scan appears to be more sensitive in detecting anomalies suggestive of peritoneal tuberculosis.

Laparoscopy is widely used for the investigation of ascites or peritoneal thickening of unknown causes [8]. It has been advocated as the ideal method for the diagnosis of peritoneal TB since it allows peritoneal inspection with the option of obtaining biopsy specimens for histopathological and microbiological evaluation (including mycobacterial culture and molecular biology) [6,13]. Laparotomy is an option in patients with extensive peritoneal adhesions, or cases where laparoscopy is not available [8].

The most common macroscopic findings are ascites, white-yellowish nodules of a uniform size (3-5 mm) on the peritoneum (also on the omentum and organs), peritoneal/visceral adhesions and the peritoneum appearing thickened, hyperaemic and sometimes haemorrhagic [8,13].

Peritoneal TB can present with a laparoscopic appearance very similar to advanced ovarian carcinoma [4]. The macroscopic appearance of peritoneal carcinomatosis usually includes peritoneal nodules with variable sizes, irregular margins, and a non-inflammatory peritoneum [9]. Sarcoidosis, starch peritonitis and Crohn’s disease may also mimic these laparoscopic features, and hence, performing peritoneal biopsies is important [6]. Despite these differences, it may not be easy to distinguish peritoneal tuberculosis from peritoneal carcinomatosis. In fact, in this case, diagnostic laparoscopy was performed and revealed lesions that were classified by the medical team as highly suggestive of disseminated peritoneal carcinomatosis. Since the definitive diagnosis is histological, biopsies were performed. Given that there was no clearly identified or suspected primary tumor, but with clinical manifestations and US findings that were quite significant (yet somewhat non-specific), samples were also collected for microbiological analysis.

Typical histologic features include caseating granulomas with epithelioid and Langerhans cells and chronic inflammatory features [13].

Gene amplification techniques, like real-time polymerase chain reaction (PCR), have shown tremendous potential. Unfortunately, it appears to have significant variability in sensitivity relative to the tissue being sampled [14]. NAAT is a useful tool, although its applicability may be limited by high costs and low sensitivity in detecting MTBC. Nevertheless, the results are quicker (24-48 hours) than cultures (it requires four to eight weeks with traditional culture media) [4,5,9].

The isolation of mycobacteria by culture (of ascitic fluid or peritoneal biopsy), ideally performed by laparoscopy, remains the gold standard diagnostic procedure [9]. Therefore, the main disadvantage of culture is the prolonged time for results, while the disadvantage of PCR tests is related to tissue-dependent sensitivity. The combination of the two techniques can be a beneficial option in centers where molecular biologic diagnostic techniques are available. The histological examination of peritoneal biopsies is also useful, namely, in the presence of granulomas and acid-fast bacilli. US or a CT scan can also be used to guide aspiration of ascitic fluid or peritoneal biopsies [5].

The treatment of peritoneal TB is primarily medical, as described previously [5]. In this case, the pelvic CT scan still presented some imaging anomalies after six months of treatment, so it was decided to extend the treatment until nine months. However, the six-month regimen of antitubercular therapy is associated with an objective clinical response in most of the patients [15]. Other forms of the disease, affecting the central nervous system and bones and joints, may require longer treatments [16]. Symptom resolution and regression of ascites are expected during treatment. Laboratory abnormalities typically normalize within three months and CA125 levels may return to normal after one to two months, making it a useful marker for monitoring the therapeutic response [9]. Surgery is unfrequently required and is indicated in patients with complications such as bowel perforation, abscess, fistula, bleeding, and/or high-grade obstruction [5].

## Conclusions

The diagnosis of peritoneal TB requires a high degree of suspicion and must be considered when evaluating a patient with non-specific abdominal symptoms, systemic involvement and ascites, particularly in young women. Laboratory findings are non-specific. When facing these clinical features, analysis of ascitic fluid and imaging tests (such as ultrasound and CT scan) can be helpful in identifying signs suggestive of peritoneal tuberculosis. However, both imaging patterns and laparoscopic features of peritoneal TB can be difficult to distinguish from peritoneal carcinomatosis. The differences may not be easy to detect as there are no major findings that differentiate them, so the definitive diagnosis will always require a biopsy. Laparoscopy with peritoneal biopsies for culture remain the gold standard diagnostic procedure when peritoneal tuberculosis is suspected, but molecular biologic diagnostic techniques are promising, providing faster diagnosis. Pharmacological treatment for six months is appropriate in most cases.
